# DNA Methylation Analysis of the *SHOX2* and *RASSF1A* Panel Using Cell-Free DNA in the Diagnosis of Malignant Pleural Effusion

**DOI:** 10.1155/2023/5888844

**Published:** 2023-01-14

**Authors:** Nana Zhang, Zichen Liu, Kun Li, Xuya Xing, Chaolian Long, Fangchao Liu, Bin She, Nanying Che

**Affiliations:** ^1^Department of Pathology, Beijing Chest Hospital, Capital Medical University, Beijing Tuberculosis and Thoracic Tumor Research Institute, Beiguandajie 9#, Tongzhou, Beijing 101149, China; ^2^Science and Technology Office, Beijing Chest Hospital, Capital Medical University, Beijing Tuberculosis and Thoracic Tumor Research Institute, Beijing, China; ^3^Academic Development, Tellgen Corporation, Shanghai, China

## Abstract

**Objectives:**

The differential diagnosis of pleural effusion (PE) is a common but major challenge in clinical practice. This study aimed to establish a strategy based on a PE-cell-free DNA (cfDNA) methylation detection system for the differential diagnosis of malignant pleural effusion (MPE) and benign pleural effusion (BPE).

**Methods:**

A total of 104 patients with PE were enrolled in this study, among which 50 patients had MPE, 9 malignant tumor patients had PE of indefinite causes, and the other 45 patients were classified as benign controls. The methylation status of short stature homeobox 2 (*SHOX2*) and RAS association domain family 1, isoform A (*RASSF1A*) was detected using PE-cfDNA specimens by real-time fluorescence quantitative PCR. Total methylation (TM) was defined as the combination of the methylation levels of *SHOX2* and *RASSF1A*. The electrochemiluminescence immunoassay was applied to evaluate the levels of multiple serum tumor markers.

**Results:**

The PE-cfDNA methylation status of either *SHOX2* or *RASSF1A* was much higher in MPE samples than in benign controls. The combination of *SHOX2* and *RASSF1A* methylation in PE yielded a diagnostic sensitivity of 96% and a specificity of 100%, respectively. When compared with the corresponding serum tumor marker detection results, TM showed the highest diagnostic efficiency (AUC = 0.985). Furthermore, the combination of the *SHOX2* and *RASSF1A* methylation panels using PE-cfDNA could apparently improve the differential diagnostic efficacy of BPE and MPE and could help compensate for the deficiency of cytology.

**Conclusions:**

Our results indicated that *SHOX2* and *RASSF1A* methylation panel detection could accurately classify BPE and MPE diseases and showed better diagnostic performance than traditional serum parameters. The *SHOX2* and *RASSF1A* methylation detection of PE-cfDNA could be a potentially effective complementary tool for cytology in the process of differential diagnosis. In summary, PE-cfDNA could be used as a promising non-invasive analyte for the auxiliary diagnosis of MPE.

## 1. Introduction

Pleural effusion (PE) is a common clinical symptom caused by over 50 different diseases, including infection, malignant tumors, heart failure, and hypoproteinemia. According to etiology, PE is typically classified as malignant pleural effusion (MPE) or benign pleural effusion (BPE) [1]. Lung cancer, metastatic breast cancer, malignant pleural mesothelioma, lymphoma, and gastrointestinal tumors are major causes of MPE, and the formation of MPE often predicts a worsening condition in patients [2]. With regard to BPE, tuberculous pleurisy is the most common cause, especially in developing countries [3]. Patients with MPE tend to have a relatively poor prognosis, and the appearance of MPE directly affects the evaluation of tumor stage and the selection of a therapeutic approach. However, patients with BPE can usually be clinically cured by means of anti-infection, anti-tuberculosis, and closed thoracic drainage treatments and have a relatively good prognosis [4]. In clinical practice, it remains a challenge to distinguish MPE and BPE, and cytology or pleural biopsy is the gold standard for diagnosing MPE [5]. Cytological examination of PE is fast, convenient, and noninvasive, with a specificity close to 100%, but the sensitivity is low due to the paucity of enough cells [6]. Pleural biopsy can provide relatively high-sensitivity, but such an invasive test is not appropriate for regular examination due to the high risk of complications [7, 8].

Classic serum tumor markers, such as carcinoembryonic antigen (CEA), neuron-specific enolase (NSE), progastrin-releasing peptide (Pro-GRP), cytokeratin fragment (CYFRA) 21-1, squamous cell carcinoma-related antigen (SCC-Ag), cancer antigen (CA) 19-9, CA-125, CA15-3, and alpha-fetoprotein (AFP), have been widely used in the identification of MPE [9–11]. However, the overall sensitivity is insufficient to meet the demand of the current clinical diagnostic efficiency. In view of the limitations of conventional detection methods, there is an urgent need to develop a more accurate and effective method for the rapid differential diagnosis of MPE.

DNA methylation is a major epigenetic mechanism that maintains gene expression and cell characteristics [12]. In particular, aberrant promoter CpG island hypermethylation may lead to the gene silencing of tumor suppressor genes, thus contributing to the occurrence of multiple cancers [13]. RAS association domain family 1, isoform A (*RASSF1A*), is a classic tumor suppressor molecule that is widely involved in signal transduction, the cell cycle, tumor metastasis and other biological pathways [14]. Short stature homeobox gene two (*SHOX2*) plays an important role in skeletal development, embryonic development, and cardiovascular differentiation. Moreover, *SHOX2* was found to regulate cell proliferation and differentiation and induce epithelial-mesenchymal transition, making it an important growth regulator in the body [15–17]. Accumulating evidence suggests that aberrant promoter CpG island hypermethylation of *SHOX2* and *RASSF1A* can serve as a diagnostic and prognostic biomarker for lung cancer, colorectal cancers, biliary tract cancers, and head and neck squamous cell carcinomas [18–21].

Cell-free DNA (cfDNA) refers to the DNA fragments released by original cells into body fluids and was first reported in human plasma in 1948 [22]. It was further discovered in multiple types of body fluids, including PE, ascites, amniotic fluid, cerebrospinal fluid, and urine, both under physiological and pathological conditions [23–26]. Currently, cfDNA has been widely used in prenatal diagnosis, tumor diagnosis, and the selection of targeted therapy management and has shown great significance in the field of liquid biopsy [27–29]. The preliminary study of our research group found that cfDNA derived from PE supernatant may serve as high-quality material for molecular detection in guiding targeted therapies [30]. Several studies have concentrated on the detection of aberrant methylation of the *SHOX2* and *RASSF1A* genes in plasma cfDNA as well as bronchoalveolar lavage fluid specimens to help improve the diagnosis of malignant tumors, while little attention has been given to cfDNA methylation in PE [31]. Compared to the traditional specimens, cfDNA from PE represents an easily accessible and direct source for the differential diagnosis of BPE and MPE and is not limited by the number of exfoliated cells.

In this study, we evaluated the *SHOX2* and *RASSF1A* promoter methylation status in definitively diagnosed PE-cfDNA from 95 patients with benign and malignant diseases using real-time fluorescence quantitative PCR technology. Furthermore, we conducted combined analysis with traditional cytology and serum tumor markers, aiming to develop a new diagnostic technique for distinguishing BPE and MPE more quickly and efficiently.

## 2. Materials and Methods

### 2.1. Clinical Specimens

The study was approved by the Human Research Ethics Committee of the Beijing Chest Hospital, Capital Medical University. 104 PE specimens were collected from consenting individuals from August 2020 to July 2021. Patient information, including sex, age, smoking history, and clinical diagnosis, was recorded. Individuals with a prior history of any cancer were excluded from this study.

### 2.2. Cytological and Pathological Analyses

PE samples from each patient were obtained by pleural puncture and chest drainage. After centrifugation at 2000 g for 10 min, the supernatant was carefully isolated into a new tube without disturbing the debris for cfDNA extraction, and the corresponding sediments were processed for liquid-based cytology or prepared into paraffin-embedded cell blocks and stained with hematoxylin and eosin. Immunocytochemical staining examination was applied for cell block sections as a supplement. All slides were individually reviewed by two experienced pathologists. The detection of pathological changes in malignant tumors in pleural biopsy tissue or cytology has been considered the diagnostic gold standard for MPE.

### 2.3. DNA Extraction and Processing

A supernatant of 5 milliliters of PE was used for DNA extraction. cfDNA was obtained using the CWhipro Circulating DNA Midi Kit (CWBIO, Jiangsu, China) following the manufacturer's instructions. The DNA yield was evaluated by the Qubit dsDNA HS Assay Kit (Life Technologies, Carlsbad, CA) on a Qubit® 3.0 fluorometer. A total of 200 ng DNA was treated with sodium bisulfite using the Tellgen DNA Purification Kit (Tellgen, Shanghai, China) to modify unmethylated cytosine to uracil. The purified bisulfite-converted DNA was directly used for methylation specific real-time PCR with the commercial LungMe® Real-time PCR kit (Tellgen, Shanghai, China). PCR was performed on a SLAN-96S platform (Hongshi, Shanghai, China) to amplify methylated *SHOX2*, *RASSF1A* and *β*-ACTB, of which the corresponding channels were VIC, FAM and CY5. The relative amount of methylation for each targeted gene was calculated using the following formula: ΔCt_*SHOX2*_ = Ct_*SHOX2*_ − Ct_*β-ACTB*_, ΔCt_*RASSF1A*_ = Ct_*RASSF1A*_ − Ct_*β-ACTB*_.

### 2.4. Tumor Marker Detection

At the clinical laboratory department, the levels of serum NSE, CEA, Pro-GRP, CYFRA 21-1, SCC-Ag, AFP, CA-125, CA15-3, and CA19-9 were detected with commercial maturity test assays (Tellgen, Shanghai, China) according to the manufacturer's instructions using a Cobas e601 analyzer. The cutoff value was set as suggested.

### 2.5. Statistical Analysis

Statistical analyses and graphics generation were performed using IBM SPSS Statistics 21.0 software (SPSS Inc., Chicago, IL) and GraphPad Prism 8.0. Descriptive statistics (GraphPad, San Diego, CA, USA) are reported as frequencies for categorical variables and medians ± interquartile ranges for continuous variables. The detection sensitivity, specificity, negative predictive value (NPV), positive predictive value (PPV), and relative 95% confidence interval (CI) for diagnosing BPE and MPE by methylation tests were calculated. The positive frequency of methylation in the *SHOX2* and *RASSF1A* genes, serum tumor marker detection, and cytology examination were analyzed using the chi-square test. The receiver operating characteristic (ROC) curve was used to determine the cutoff values of ΔCt_*SHOX2*_ and ΔCt_*RASSF1A*_ and to calculate the area under the ROC curve (AUC), aiming to evaluate the diagnostic efficacy. A two-sided *p* value <0.05 was considered to be statistically significant.

## 3. Results

### 3.1. Characteristics of the Patients

A total of 104 patients with symptomatic PE were finally enrolled in this study. There were 67 males and 37 females, with a mean age of 57.9 years (range: 19∼82). Fifty-nine cases were diagnosed as definite malignant tumors by histopathology or cytology analysis, including 52 lung cancers, 2 thymic squamous cell carcinomas, 1 ovarian cancer, 1 lymphoma, 1 mesothelioma, 1 adenoid cystadenocarcinoma, and 1 esophageal cancer. Among the cohort, 50 patients were determined to have MPE, while 9 patients had pleural effusions of indefinite causes. The other 45 patients were classified as benign controls, comprising 37 patients with tuberculosis, 5 patients with pulmonary infections, and 3 patients with heart failure diseases. All the demographic and clinical characteristics are summarized in Table 1.

### 3.2. Diagnostic Performance of the cfDNA Methylation Test for BPE and MPE

**Figure 1:**
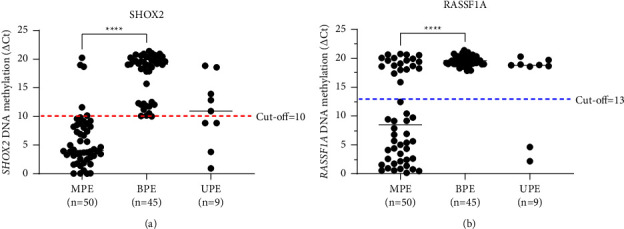
Quantitative analysis of *SHOX2* (a) and *RASSF1A* (b) DNA methylation in BPE and MPE specimens. (a) *SHOX2* DNA methylation in 50 MPE, 45 BPE, and 9 UPE samples. The red dotted line indicates the cutoff value of *SHOX2*. (b) *RASSF1A* DNA methylation in 50 MPE, 45 BPE, and 9 UPE samples. The blue dotted line indicates the cutoff value of *RASSF1A*. ^*∗∗∗∗*^*p* < 0.0001.

**Figure 2:**
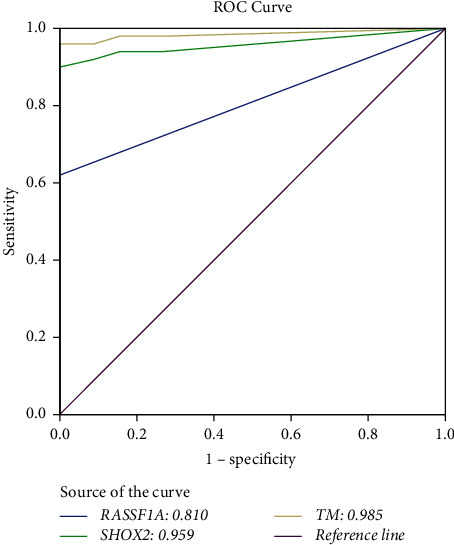
ROC curve for the diagnostic value of *SHOX2* and *RASSF1A* and TM.

We evaluated the diagnostic value of *SHOX2* and *RASSF1A* methylation by real-time PCR using PE-cfDNA specimens. Ct_*SHOX2*_ <32 and Ct_*RASSF1A*_ <35 criteria were set for calculating the delta cycle threshold (ΔCt) [32]. We assigned “NoCt” = 40. The quantitative *SHOX2* and *RASSF1A* methylation status (ΔCt_*SHOX2*_ and ΔCt_*RASSF1A*_) of the tested specimens is plotted in detail in Figure 1. The methylation level of *SHOX2* was much higher in MPE samples than in benign controls (A, 5.7 ± 4.6 vs. 17.4 ± 3.8, *p* < 0.0001), and the same tendency was observed in the RASSF1A (B, 10.0 ± 7.8 vs. 19.6 ± 0.9, *p* < 0.0001). According to the ΔCt method, the cutoff value of *SHOX2* and *RASSF1A* methylation was defined as the maximum of Youden index. ROC analysis was performed, where the AUC was calculated and the cutoff value was determined accordingly. As shown in Figure 2, the AUCs of methylation *SHOX2*, methylation *RASSF1A* and the combination of *SHOX2* and *RASSF1A* (TM, total methylation) were 0.810, 0.959, and 0.985, respectively, which showed good discriminative power in differentiating BPE from MPE. The optimal cutoff points of *SHOX2* and *RASSF1A* methylation in this study were set at ΔCt_*SHOX2*_ = 10.0 and ΔCt_*RASSF1A*_ = 13.0. Based on this cutoff, the detection sensitivities of *SHOX2* and *RASSF1A* were 90.0% and 62.0%, respectively, while combination analysis of these two genes improved the sensitivity up to 96.0%, which was higher than that of any single parameter, while the specificity remained unchanged at a high level of 100% (Table 2). These results showed that the methylation levels of *SHOX2* and *RASSF1A* using PE-cfDNA had potential diagnostic value in the differentiation of BPE and MPE, while the combination panel of *SHOX2* and *RASSF1A* had the best diagnostic efficiency.

### 3.3. Clinicopathological Correlation Analysis

We further tested whether PE-cfDNA methylation levels were associated with the corresponding clinicopathological parameters. As suggested in Table 3, the methylation level of *SHOX2* was higher in elderly patients (*p*=0.018), while there was no significant association of the *RASSF1A* methylation level or TM with patient age, sex or smoking status. In our study cohort, lung cancer (nonsmall-cell lung cancer, NSCLC and small cell lung cancer, SCLC) accounted for the largest proportion of MPE, and the sensitivity of *SHOX2*, *RASSF1A*, and TM were 91.1% (41/45), 64.4% (29/45), and 97.8% (44/45), respectively. A total of 42 NSCLC (37 lung adenocarcinomas, 3 lung squamous carcinomas, and 2 indefinite type) and 3 SCLC patients were included in the lung cancer group. Both NSCLC and SCLC-derived MPE showed relatively high methylation positive rates of 97.6% (41/42) and 100% (3/3), respectively. In lung adenocarcinoma, the methylation positive rates of *SHOX2*, *RASSF1A*, and TM were 91.9% (34/37), 59.5% (22/37), and 97.3% (36/37), respectively. Tuberculosis diseases accounted for a great proportion of BPM cases, showing an excellent methylation detection specificity of 100%. More cases derived from multiple malignant or benign diseases are needed in future studies.

### 3.4. Comparison of cfDNA Methylation Detection with Traditional Analysis

**Figure 3:**
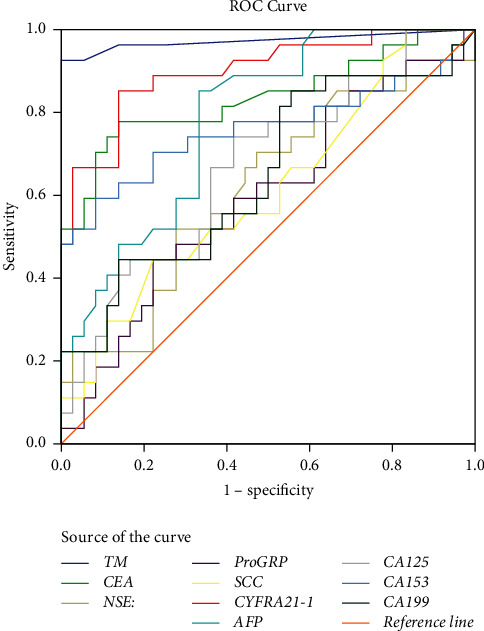
ROC curves for the diagnostic value of TM, CEA, NSE, pro-GRP, SCC, CYFRA21-1, AFP, CA-125, CA15-3, and CA19-9.

We next compared the diagnostic efficacy of the combined *SHOX2* and *RASSF1A* methylation panels with that of multiple traditional serum tumor markers. As shown in Figure 3 and Table 4, the AUC of TM was significantly higher than those of each of the traditional serum tumor markers. In contrast, the detection sensitivity and specificity of traditional serum tumor markers were relatively low, among which CA-125 exhibited the highest sensitivity of 78.6% while the specificity was only 47.2%. TM detection achieved a sensitivity of 96% (95% CI: 78.8%–95.9%) and a specificity of 100% (95% CI: 90.2%–100%), obviously exceeding those of the above methods.

Moreover, in our cohort, 68.0% (34/50) of MPE cases had definite positive cell block cytology, 8% (4/50) were atypical, and 24% (12/50) were negative. In contrast, liquid-based cytology was definitively positive in 54.1% (20/37) of MPE cases, atypia in 16.2% (6/37) of the cases, and negative in 29.7% (11/37) of the cases. A positive result was recorded when cancer cells were found by either of the above two methods. When they were combined, the positive rate of cytology increased to 74% (37/50), the atypia rate was 10% (5/50), and the negative rate was 16% (8/50). The positive rate of the PE-cfDNA methylation assay for *SHOX2* and *RASSF1A* was 94.6% (35/37) in the MPE group with definite positive cytology, and 100% (5/5) and 100% (8/8) in the MPE group with atypia and negative cytology (further confirmed by cytology or pleural biopsy during the same period), respectively. The combined methylation detection of *SHOX2* and *RASSF1A* in PE-cfDNA yielded a diagnostic sensitivity of up to 96%, suggesting that it could be a potential effective complementary tool for cytology in the differential diagnosis of MPE and BPE. In summary, the use of PE-cfDNA methylation of *SHOX2* and *RASSF1A* could facilitate the early diagnosis and differential diagnosis of MPE.

## 4. Discussion

MPE, as a common symptom of metastatic lung cancer, breast cancer, and other cancer-related pleural lesions, upgrades the tumor stage and precludes resection of the primary tumor, indicating a poor prognosis for the patient. Nevertheless, patients with BPE can usually be clinically cured if treated in a timely manner. Hence, it is of great significance to accurately differentiate between BPE and MPE for therapeutic decisions and thus improve the prognosis of patients with MPE. However, to date, no specific method for differential diagnosis is readily available in clinical practice due to either its low sensitivity or specificity. Thoracoscopy exhibited an excellent diagnostic yield (>95%) for malignant pleural lesions and was considered the gold standard. However, high requirements for surgical staff and equipment, as well as high complications, limit its application [33]. Cytology analysis can identify malignancy in approximately 60% of patients but easily causes misdiagnosis [34]. In terms of morphologic imaging, CT and FDG-PET showed a potential role in identifying the nature of PE, but the false-positive rate was relatively high. When combination analysis was used, the specificity, PPV, and accuracy reached 76%, 67%, and 84%, respectively [35]. The test results of emerging molecular detection technologies are more sensitive and objective, which can compensate for the shortcomings of morphological diagnosis.

Aberrant DNA methylation is correlated with the malignant characteristics of various tumors [36]. As one of the most promising new biomarkers in the diagnosis of cancer, aberrant methylation of a set of genes, including *SHOX2*, *RASSF1A*, and *SEPT9*, has been transferred into clinical application [37–39]. The association of cancer diagnosis with the abnormal methylation status of the *SHOX2* and *RASSF1A* genes has been studied in multiple sample types, including bronchoalveolar lavage fluid and formalin-fixed paraffin-embedded (FFPE) tissues. The combination of *SHOX2* and *RASSF1A* methylation in bronchoalveolar lavage fluid yielded a diagnostic sensitivity of 81.0% and specificity of 97.4%, showing the highest diagnostic efficiency compared with the established cytology examination and serum biomarker CEA [40]. Based on FFPE samples, the positive detection rates of the *SHOX2* and *RASSF1A* panels in SCLC, SCC, and adenocarcinomas were 100%, 96.1%, and 82.9%, respectively. Furthermore, *SHOX2* methylation, but not *RASSF1A* methylation, was correlated with the stages of lung cancer [32]. As promising markers of DNA methylation for the diagnosis of thoracic malignancies, few studies using PE-cfDNA have been conducted. In the field of tumor targeted therapy, cfDNA derived from PE supernatant was reported as a high-quality material for molecular detection, which was readily available and less affected by the amount of cells shed [30].

In this study, we evaluated the promoter methylation status of *SHOX2* and *RASSF1A* using PE-cfDNA in 104 PE cases with the Methylated Human *SHOX2* and *RASSF1A* Gene Detection Kit (Tellgen Co. Ltd., Shanghai, China). The results showed that the methylation levels of *SHOX2* and *RASSF1A* were obviously higher in the MPE group than in the benign control group, which was in accordance with our understanding that hypermethylation is associated with malignancy. ROC curves were plotted to evaluate the diagnostic ability of *SHOX2* and *RASSF1A* DNA methylation in PE, with AUC values of 0.959 for *SHOX2*, 0.810 for *RASSF1A*, and 0.985 for the combination analysis. This indicated that the methylation of *SHOX2* or *RASSF1A* demonstrated an efficient diagnostic ability to distinguish MPE from BPE, with the combination performing best. Based on the calculated cutoff values (ΔCt_*SHOX2*_ = 10.0 and ΔCt_*RASSF1A*_ = 13.0), we found that the methylation positive rates of *SHOX2* and *RASSF1A* were obviously higher in the MPE group (90.0% and 62.0%) than in the benign control group (0% and 0%) and the no-exact-diagnosis group (44.4% and 22.2%). The combination of *SHOX2* and *RASSF1A* greatly enhanced the detection positivity rate to 96%, with a high specificity of 100%. Multiple potential diseases causing PE were included in our cohort, but a certain group had a limited number of samples. Lung cancer was the major cause of MPE (45/50). The detection sensitivities of *SHOX2*, *RASSF1A*, and TM in lung cancer-derived MPE were 91.1%, 64.4%, and 97.8%, slightly higher than those in the overall MPE samples. In lung adenocarcinoma-derived MPE, the methylation positive rates were 91.9% for *SHOX2* and 59.5% for *RASSF1A*, which were different from those in a previous study using histology specimens, 54.9% for *SHOX2* and 46.3% for *RASSF1A* in adenocarcinoma [18]. In three lung squamous cell carcinoma-derived MPE patients, both *SHOX2* and *RASSF1A* methylation detections were positive. There have also been studies about the distribution of the methylation status of *SHOX2* and *RASSF1A* in different lung cancer subtypes using bronchial aspirates and FFPE samples. The data showed that the positive detection rate of *SHOX2* in SCLC was 80–100%, followed by a percentage of 63–96.1% in squamous cell carcinoma, while adenocarcinomas exhibited the lowest positive detection rate of 39–82.9% [41, 42]. *RASSF1A* methylation was more frequently detected in adenocarcinomas (39%) than in squamous cell carcinomas (13%) [43]. In our cohort, MPE was principally adenocarcinoma-derived, and whether the methylation positive rates of *SHOX2* and *RASSF1A* are different in specific disease subtypes needs further exploration. Moreover, we applied the methylation test using PE-cfDNA and obtained ideal diagnostic results. A comparison between PE-cfDNA and the cell pellet samples will be performed in the future. In addition, four of the other five MPE patients were positive, with single positivity for *SHOX2* in ovarian and thymic squamous cell carcinoma and double positivity for *SHOX2* and *RASSF1A* in lymphoma and adenoid cystic carcinoma. *SHOX2* and *RASSF1A* methylation were not detected in mesothelioma, but individual samples were not representative. A larger sample size for each disease is needed to validate the results in the future. Overall, the positive detection results suggested the potential for the wide use of methylation detection in multiple malignancy-derived PE samples.

Several common serum biomarkers, such as NSE, CEA, and CYFRA 21-1, have been widely used in clinical practice [44]. In comparison with the conventional assays, the methylation analysis of the *SHOX2* and *RASSF1A* panels using PE-cfDNA showed the best diagnostic power, with an AUC of 0.985. Furthermore, we found that it presents some advantages over traditional noninvasive cytological testing and invasive thoracoscopic as well as pleural biopsy techniques. In our study, of the thirteen MPE samples detected with atypical and negative cytology, the positive rate for combined *SHOX2* and *RASSF1A* methylation assays using cfDNA reached 100%, which can substantially improve the diagnostic sensitivity of cytology. As a noninvasive detection method, it can be applied to most patients with PE and can provide an important basis for the determination of PE traits.

There were some limitations in the present study. First, the sample size was relatively small. Studies with a larger number of patients need to be conducted to validate the results in the future. Second, the MPE samples collected in the study were mainly derived from lung cancer patients, and more disease types should be included. Furthermore, whether the methylation changes of the two genes were correlated with the prognosis of patients is not clear.

In summary, the methylation analysis of the *SHOX2* and *RASSF1A* panels using cf-DNA showed efficient diagnostic ability in differentiating MPE from BPE and could be a potential tool to greatly enhance the results. It may have the potential to be a complementary tool for cytology in the differential diagnosis of MPE.

## Figures and Tables

**Table 1:** Characteristics of all subjects.

	Total	Malignant	Nonmalignant
Age (years)			
Mean ± SD	57.9 ± 15.2	61.6 ± 9.3	53.0 ± 19.6
Range	19∼82	33∼82	19∼82
Gender			
Male (*n*, %)	67 (64.4%)	36 (61.0%)	31 (68.9%)
Female (*n*, %)	37 (35.6%)	23 (39.0%)	14 (31.1%)
Smoking habit			
Smoker (*n*, %)	45 (43.3%)	31 (52.5%)	14 (31.1%)
Nonsmoker (*n*, %)	59 (56.7%)	28 (47.5%)	31 (68.9%)
MPE			
Lung cancer	—	45	—
Ovary cancer	—	1	—
Thymic squamous cell carcinoma	—	1	—
Lymphoma	—	1	—
Mesothelioma	—	1	—
Adenoid cystadenocarcinoma	—	1	—
BPE			
Tuberculosis	—	—	37
Pulmonary infection	—	—	5
Heart failure	—	—	3
UPE			
Lung cancer		7	
Esophagus cancer		1	
Thymic squamous cell carcinoma		1	

MPE = malignant pleural effusion; BPE = benign pleural effusion; UPE = pleural effusion with undefinite diagnosis.

**Table 2:** Performance of the PE-cfDNA methylation test for BPE and MPE.

	Sensitivity (%)		Specificity (%)		NPV (%)		PPV (%)		Cut-off score
	Value	95% CI	Value	95% CI	Value	95% CI	Value	95% CI	
*SHOX2*	90.0	77.4–96.3	100.0	90.2–100.0	90.0	77.4–96.3	100.0	90.2–100.0	10.0
*RASSF1A*	62.0	47.2–75.0	100.0	90.2–100.0	70.3	57.4–80.8	100.0	86.3–100.0	13.0
TM	96.0	85.1–99.3	100.0	90.2–100.0	95.7	84.3–99.3	100.0	90.8–100.0	—

*SHOX2*: short stature homeobox 2; *RASSF1A*: RAS-associated domain family 1 subtype A. CI: confidence interval; PPV, positive predictive value; NPV, negative predictive value; and TM, total methylation.

**Table 3:** Univariate analysis of clinical variables and malignant effusion.

Variables	*n*	*SHOX2* methylation		*p* value	*RASSF1A* methylation		*p* value	Total methylation		*p* value
		Negative	Positive		Negative	Positive		Negative	Positive	
Age (years)										
≤50	5	2	3	**0.018**	1	4	>0.05	0	5	>0.05
>50	45	3	42		18	27		2	43	
Sex										
Male	27	3	24	>0.05	8	19	>0.05	1	26	>0.05
Female	23	2	21		11	12		1	22	
Smoking habit										
Smoker	24	1	23	>0.05	6	18	>0.05	0	24	>0.05
Nonsmoker	26	4	22		13	13		2	24	
MPE										
Lung cancer	45									
NSCLC	42	4	38	—	16	26	—	1	41	—
SCLC	3	0	3		0	3		0	3	
Ovarian cancer	1	0	1	—	1	0	—	0	1	—
Thymic squamous cell carcinoma	1	0	1	—	1	0	—	0	1	—
Lymphoma	1	0	1	—	0	1	—	0	1	—
Mesothelioma	1	1	0	—	1	0	—	1	0	—
Adenoid cystadenocarcinoma	1	0	1	—	0	1	—	0	1	—
BPE										
Tuberculosis	37	37	0	—	37	0	—	37	0	—
Pulmonary infection	5	5	0	—	5	0	—	5	0	—
Heart failure	3	3	0	—	3	0	—	3	0	—

**Table 4:** Diagnostic performance of different markers.

	Sensitivity% (95% CI)	Specificity% (95% CI)	NPV% (95% CI)	PPV% (95% CI)	Cut-off score
Total methylation	96.0 (78.8–95.9)	100.0 (90.2–100.0)	83.3 (70.2–91.6)	100.0 (91.3–100.0)	—
CEA (ng/mL)	48.9 (33.9–64.0)	100.0 (89.8–100.0)	65.2 (52.3–76.2)	100.0 (81.5–100.0)	6
NSE (ng/mL)	17.8 (8.5–32.6)	100.0 (89.8–100.0)	53.8 (42.3–64.8)	100.0 (59.8–100.0)	40
Pro-GRP (ng/mL)	11.1 (4.2–24.8)	88.4 (74.1–95.6)	48.7 (37.3–60.2)	50.0 (20.1–79.9)	80
SCC (ng/mL)	0 (0–9.8)	100.0 (89.8–100.0)	48.9 (38.1–59.7)	—	4
CYFRA21-1 (ng/mL)	42.2 (28.0–57.8)	97.7 (86.2–99.9)	61.8 (49.1–73.0)	95.0 (73.1–99.7)	6
AFP (IU/mL)	0 (0–15.0)	100.0 (88.0–100.0)	56.3 (43.3–68.4)	—	5.8
CA-125 (IU/mL)	78.6 (58.5–91.0)	47.2 (30.8–64.3)	73.9 (51.3–88.9)	53.7 (37.6–69.0)	35
CA-153 (IU/mL)	46.4 (28.0–65.8)	100.0 (88.0–100.0)	70.6 (56.0–82.1)	100 (71.7–100)	34.5
CA-199 (IU/mL)	21.4 (9.0–41.5)	97.2 (83.8–99.9)	61.4 (47.6–73.7)	85.7 (42.0–99.2)	39

CI, confidence interval; PPV, positive predictive value; NPV, negative predictive value.

## Data Availability

Some or all data generated or used during the study can be obtained from the corresponding author upon request.
